# *BRCA1* and *BRCA2* Variation in Taiwanese General Population and the Cancer Cohort

**DOI:** 10.3389/fmolb.2021.685174

**Published:** 2021-06-21

**Authors:** Jiasheng Chian, Siddharth Sinha, Zixin Qin, San Ming Wang

**Affiliations:** Cancer Centre and Institute of Translational Medicine, Faculty of Health Sciences, University of Macau, Macau, China

**Keywords:** BRCA1, BRCA2, prevalence, population, ethnicity, pathogenic

## Abstract

*BRCA1* and *BRCA2* (*BRCA*) play essential roles in maintaining genome stability. Rapidly evolving human *BRCA* generates oncogenic variants causing high cancer risk. *BRCA* variation is ethnic-specific in reflecting adaptation and/or effects of genetic drift. Taiwanese population of 23.8 million is an admixture of multiple ethnic origins; Taiwan’s subtropical and tropical climate and geographically islandic location provide a unique natural environment. Therefore, Taiwanese population provides a unique model to study human *BRCA* variation. Through collecting, standardizing, annotating, and classifying publicly available *BRCA* variants derived from Taiwanese general population and the cancer cohort, we identified 335 *BRCA* variants, of which 164 were from 1,517 non-cancer individuals, 126 from 2,665 cancer individuals, and 45 from both types of individuals. We compared the variant data with those from other ethnic populations such as mainland Chinese, Macau Chinese, Japanese, Korean, Indian, and non-Asians. We observed that the sharing rates with other Asian ethnic populations were correlated with its genetic relationship. Over 60% of the 335 Taiwanese *BRCA* variants were VUS, unclassified variants, or novel variants, reflecting the ethnic-specific features of Taiwanese *BRCA* variation. While it remains challenging to classify these variants, our structural and *in silico* analyses predicted their enrichment of *BRCA* deleterious variants. We further determined the 3.8% prevalence of *BRCA* pathogenic variants in the Taiwanese breast cancer cohort, and determined 0.53% prevalence of the *BRCA* pathogenic variants in Taiwanese general population, with the estimated 126,140 *BRCA* pathogenic variant carriers. We identified *BRCA2* c.5164_5165delAG at *BRCA2* BRC6 motif as a potential founder mutation in Taiwanese population. Our study on *BRCA* variation in Taiwanese and other East Asian populations demonstrates that ethnic specificity is a common phenomenon for *BRCA* variation in East Asian population; the data generated from the study provide a reference for clinical applications in *BRCA*-related cancer in Taiwanese population.

## Introduction

*BRCA1* and *BRCA2* (hereafter refer as *BRCA*) play essential roles in maintaining genome stability by repairing double-strand DNA damage through homologous recombination (Roy et al., 2011). *BRCA* is under positive selection in the humans, leading to high variability (Lou et al., 2014). While the majority of variants can be beneficial or neutral, those occurred at specific positions can damage the function of *BRCA*, causing genome instability and increased risk of breast cancer, ovarian cancer, and other types of cancer (Kuchenbaecker et al., 2017). As *BRCA* variation is mostly of the germline nature, the later life stage of cancer occurrence provides a unique opportunity to prevent *BRCA* variation–caused cancer by early identification of the pathogenic variant carriers before cancer development (Burke et al., 1997). Furthermore, PARP inhibitors provide effective treatment of *BRCA* variant–caused cancer through synthetic lethal therapy (Jerez et al., 2020).

**FIGURE 1:**
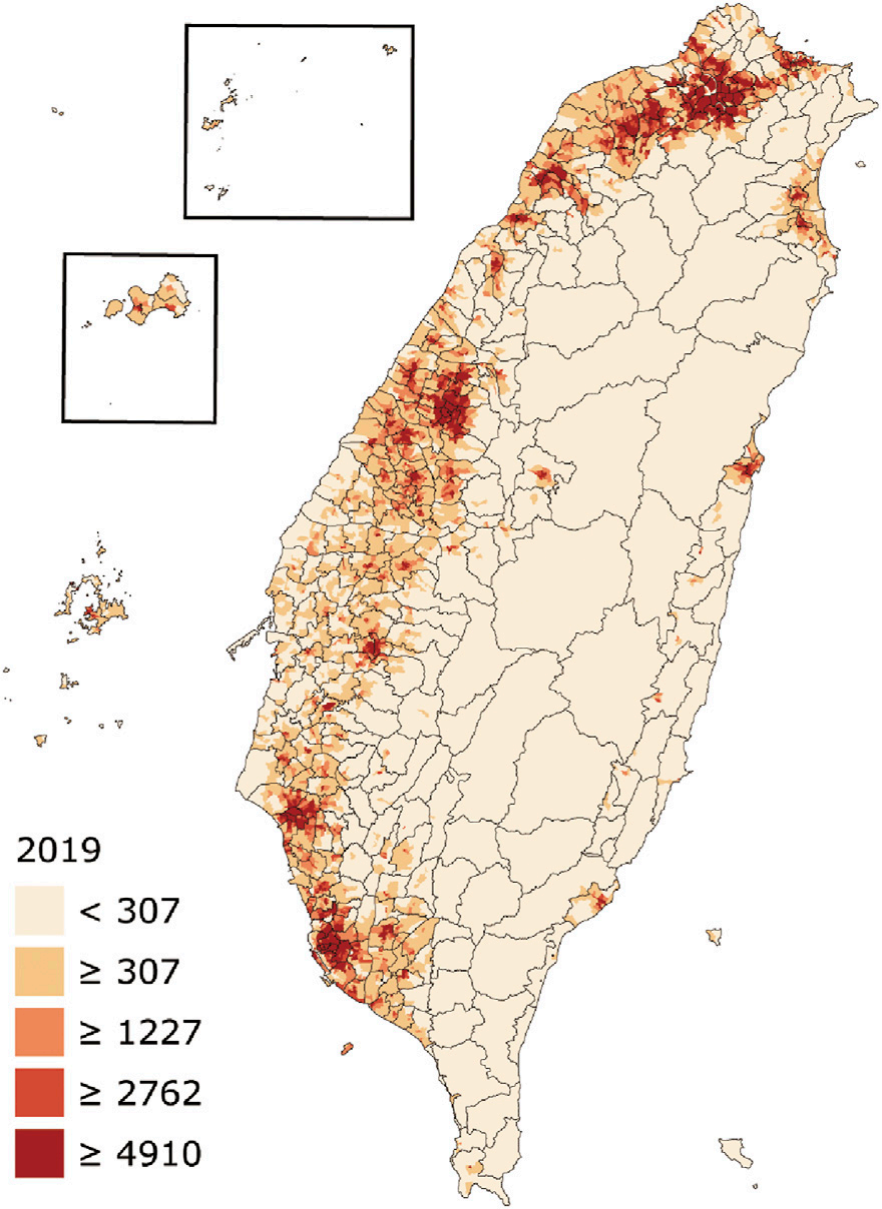
Geographic map and population density of Taiwan. The numbers show residents per square kilometer by village (from: https://en.wikipedia.org/wiki/Demographics_of_Taiwan).

*BRCA* variation is well determined as highly ethnic specific in certain ethnic populations, such as the *BRCA1* 185delAG, 5382insC, and *BRCA2* 6174delT in Ashkenazi Jews population (Levy-Lahad et al., 1997). Restricted by the lack of *BRCA* variation data from non-Caucasian populations (Bhaskaran et al., 2019; Friebel et al., 2019), however, it remains unclear whether ethnic specificity is mainly in certain specific ethnic population or is a universal phenomenon across worldwide ethnic populations. Recently, we analyzed *BRCA* variation in Asian populations such as Indian, Chinese, Korean, and Japanese, and revealed that ethnic-specific *BRCA* variation is also widely present in these Asian populations (Bhaskaran et al., 2020). With a population size nearly 24 million, Taiwanese population consists of admixed ethnic origins across prehistory and current days. Although Taiwanese population included largely the ancestors from southern Han Chinese of Fujian and Guangdong regions of mainland China, it also included other ethnicities including the native Austronesians who also distributed to Pacific islands and Asian neighbors. Furthermore, the islandic location with subtropical and tropical climates in Taiwan Island provides a unique natural environment for Taiwanese population (Chen et al., 2016; Figure 1). Therefore, the Taiwanese population provides a unique model to study *BRCA* evolution and its impact on human health.

In the current study, we performed a systematic analysis for *BRCA* variation in the Taiwanese general population and the cancer cohort. Of the *BRCA* variants identified, we observed that forty percent *BRCA* variants were Taiwanese specific; using the identified *BRCA* pathogenic variants as the reference, we determined the prevalence of *BRCA* pathogenic variation in Taiwanese general population and the cancer cohort. Data from our study provide further evidence to demonstrate that ethnic specificity of *BRCA* variation is a common phenomenon in East Asian populations.

## Results

### Data Collection

We collected a total of 335 *BRCA* variants derived from Taiwanese population, including 164 from general population, 126 from the Taiwanese cancer patient cohort, and 45 (19 in *BRCA1* and 26 in *BRCA2*) from both groups. For the variants from cancer patients, nearly all were from breast cancer and ovarian cancer (Supplementary Table S1 **)**. We performed standardization, annotation, and clinical classification for all *BRCA* variants (Table 1, Supplementary Tables S2, S3).

**TABLE 1:** BRCA variants identified in Taiwanese population.

Mutation type	General population		Total (%)	Cancer population		Total (%)	*p* Value
	*BRCA1* (%)	*BRCA2* (%)		*BRCA1* (%)	*BRCA2* (%)	—	—
A. Types of variation							
Nonsynonymous SNV	43 (53.1)	71 (55.5)	114 (54.5)	21 (31.3)	42 (40.4)	63 (36.8)	***0.001***
Synonymous SNV	27 (33.3)	40 (31.3)	67 (32.1)	8 (11.9)	11 (10.6)	19 (11.1)	***0.000***
Intron variant	7 (8.6)	10 (7.8)	17 (8.1)	7 (10.4)	2 (1.9)	9 (5.3)	0.270
Stopgain/nonsense	0 (0)	1 (0.8)	1 (0.5)	8 (11.9)	11 (10.6)	19 (11.1)	***0.000***
Frameshift deletion	2 (2.5)	4 (3.1)	6 (2.9)	19 (28.4)	28 (26.9)	47 (27.5)	***0.000***
Frameshift insertion	1 (1.2)	1 (0.8)	2 (1.0)	1 (1.5)	7 (6.7)	8 (4.7)	***0.024***
Non-frameshift deletion	1 (1.2)	1 (0.8)	2 (1.0)	0 (0)	1 (1.0)	1 (0.6)	0.683
5’/3’ UTR	0 (0)	0 (0)	0 (0)	1 (1.5)	0 (0)	1 (0.6)	0.268
Splice site	0 (0)	0 (0)	0 (0)	2 (3.0)	2 (1.9)	4 (2.3)	***0.026***
B. Clinical classification							
Pathogenic	1 (1.2)	6 (4.7)	7 (3.3)	28 (41.8)	42 (40.4)	70 (40.9)	***0.000***
Likely pathogenic	1 (1.2)	0 (0)	1 (0.5)	3 (4.5)	1 (1.0)	4 (2.3)	0.113
Uncertain significance	20 (24.7)	32 (25)	52 (24.9)	9 (13.4)	15 (14.4)	24 (14.0)	***0.009***
Likely benign	34 (42)	49 (38.3)	83 (39.7)	9 (13.4)	20 (19.2)	29 (17.0)	***0.000***
Benign	20 (24.7)	35 (27.3)	55 (26.3)	13 (19.4)	18 (17.3)	31 (18.1)	0.058
Conflicting interpretations	2 (2.5)	2 (1.6)	4 (1.9)	0 (0)	0 (0)	0 (0)	0.069
Unclassified	3 (3.7)	4 (3.1)	7 (3.3)	5 (7.5)	8 (7.7)	13 (7.6)	0.065
Total	81	128	209	67	104	171	—

The bold-italic values indicate the numbers between general population and cancer population were statistically significant different.

### Similarity and Differences Between General Population and the Cancer Cohort

Data from both general population and cancer patients gave a unique opportunity to compare the similarity and differences of *BRCA* variation between the two groups with the same ethnic background. Although the total number of *BRCA* variants at the individual level was similar, significant differences existed between the two groups. The types of *BRCA* variation between the two groups were significantly different, including nonsynonymous SNV, synonymous SNV, stopgain, frameshift deletion, frameshift insertion, and splice site; and the frequency of nonsynonymous SNV and synonymous SNV in general population was higher than that in the cancer cohort (54.5% vs. 36.8% and 32.1% vs. 11.1%, *p* < 0.001 and 0.000, accordingly), whereas the frequency of stopgain, frameshift deletion/insertion, and splice variants was higher in the cancer cohort than that in the general population (Table 1). Significant differences in the clinical classification were also present in between. For example, 40.9% of *BRCA* variants in the cancer cohort were pathogenic variants, which was much higher than the value of 3.3% in general population (*p* < 0.000); VUS (variants of uncertain significance) and likely benign were significantly higher in general population than those in the cancer cohort (24.9% vs. 14% and 39.7% vs. 17%, *p* < 0.009, 0.000 accordingly) (Table 1).

### Similarity and Differences From Other Ethnic Populations

We compared *BRCA* variants between Taiwanese population and other populations including mainland Chinese (Bhaskaran et al., 2019); Macau Chinese representing southern Chinese (Qin et al., 2020); Asian populations including Korean, Japanese, and Indian (Bhaskaran et al., 2020); and non-Asian populations, of which the majority were Caucasians (Dutil et al., 2015; Rebbeck et al., 2018). The results show that 35.5 and 37.6% of the Taiwanese variants were shared with Macau Chinese and mainland Chinese, respectively, 27.5% with Japanese, 20.3% with Korean, 11.3% with Indian, and 53.1% of entire non-Asian populations. The different sharing rates reflected the evolutionary relationship of Taiwanese population with non-Taiwanese populations (Table 2). We also compared with the *BRCA* variants from Fujian Chinese, which has the closest genetic tie with the Taiwanese population. Of the 18 *BRCA* variants available for comparison, 8 (44.4%) were matched by Taiwanese variants.

**TABLE 2:** Comparison between Taiwanese population and other populations.

Population	*BRCA1*	Matched	*BRCA2*	Matched	*BRCA*	Matched (%)	Unmatched (%)
	Total		Total		Total		
Chinese							
Mainland Chinese	758	57	764	69	1,522	126 (37.6)	209 (62.4)
Macau Chinese	264	46	395	73	659	119 (35.5)	216 (64.5)
Non-Chinese Asian							
Japanese	415	37	649	55	1,064	92 (27.5)	243 (72.5)
Korean	281	35	300	33	581	68 (20.3)	267 (79.7)
Indian	274	23	244	15	518	38 (11.3)	297 (88.7)
Non-Asian							
BED	19,190	71	19,906	107	39,096	178 (53.1)	157 (46.9)
Total^a^	—	84		133	—	217 (64.8)	118 (35.2)

a Distinct numbers by counting overlapped variants only once.

### VUS, Unclassified Variants, and Novel Variants

Of the *BRCA* variants identified, 20.7% were VUS (52 in *BRCA1* and 24 in *BRCA2*, Table 1), 6.0% were unclassified variants (seven in *BRCA1* and 13 in *BRCA2*, Table 1), and 35.2% (118 *BRCA* variants) were absent in the *BRCA* data from worldwide ethnic populations (Table 2 **)**. The combination of VUS, unclassified, and novel variants accounted for 61.9% of all 335 *BRCA* variants identified in the Taiwanese population. Although the definitive classification for these variants remains to be solved, they may enrich with the Taiwanese-specific pathogenic *BRCA* variants. For example, 64.4% of the 118 *BRCA* variants were nonsynonymous SNV, frameshift insertion/deletion/substitution, stopgain, and non-frameshift deletion (Table 2, Supplementary Tables S2, S3).

**FIGURE 2:**
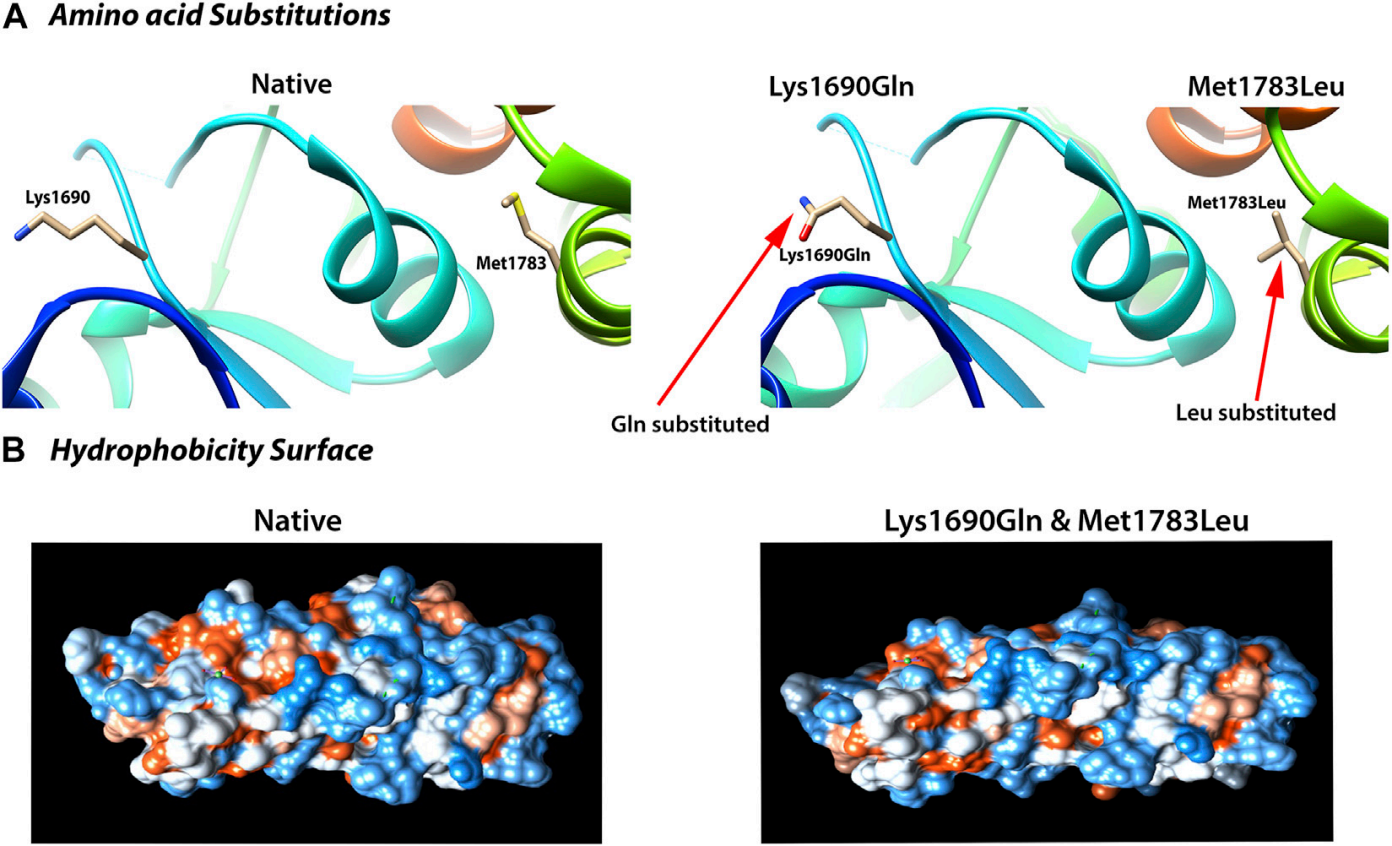
Deleterious impact of unclassified variants (c.5068 A > C; p.Lys1690Gln; c.5347 A > C; p.Met1783Leu) on BRCA1 BRCT structural stability. **(A)** Amino acid substitution showing the variant-caused amino acid change from Lys and Met (left) in the native structure to Gln and Leu (right) at the position of 1,690 and 1783, respectively. **(B)** Deleterious effects reflected by the change in hydrophobicity surface in the mutant BRCT. Both Lys1690Gln and Met1783Leu caused nearly identical change as shown here. The results were from 40 ns simulation (see text for detailed explanation).

To further test this possibility, we used the molecular dynamic simulation (MDS) to measure the impact of the four *BRCA1* unclassified variants (c.5068A > C p.Lys1690Gln, c.5347A > C p.Met1783Leu; c.5347A > G p.Met1783Val; c.5349G > A p.Met1783Ile) located at *BRCA1* BRCT repeat on BRCT structural stability, and use the information as the indication for their potential deleterious effects. Of the four unclassified variants, c.5068A > C p.Lys1690Gln and c.5347A > C p.Met1783Leu were predicted to be deleterious (Figure 2). Taking c.5347A > C p.Met1783Leu as an example, p.Met1783 is located within the α’1 helix at C terminal near the edge of the inter repeat interface of the native BRCT structure. While p.Met1783Leu by c.5347A > C was sterically stable without physical contact or clashes with adjoining residues, it unfolded the structure of BRCT and destabilized the hydrophobic interface, causing reposition between the two *BRCA1* BRCT repeats as reflected by the larger structure deviation and flexibility, reduced NH bond, and decreased structure compactness as measured by six different MDS programs (RMSD, RMSF, Rg, SASA, NH bond, and Covariance). The results showed that of the three missense variant-caused substitutions at the same position (p.Met1783Leu; p.Met1783Val; p.Met1783Ile), p.Met1783Leu was deleterious by disturbing BRCT structure stability.

We also used four different types of *in silico* prediction programs including SIFT, Polyphen2, LRT, and MutationTaster to predict the deleteriousness of the four unclassified variants. The results showed that the two deleterious variants (p.Lys1690Gln, p.Met1783Leu) predicted by MDS were also predicted as deleterious by at least three different programs. For example, p.Met1783Leu was predicted by all four programs as deleterious (Table 3).

**TABLE 3:** Prediction of deleterious variants for the unclassified variants in BRCA1 BRCT repeats.

cDNA	Amino acid	MDS	*In silico* prediction programs				
			SIFT	Polyphen2_HDIV	LRT	MutationTaster	Total deleterious
c.5068A > C	p.Lys1690Gln	Deleterious	Deleterious	Probably damaging	Neutral	Disease causing	3 of 4
c.5347A > C	p.Met1783Leu	Deleterious	Deleterious	Probably damaging	Deleterious	Disease causing	4 of 4
c.5347A > G	p.Met1783Val	Tolerated	Deleterious	Probably damaging	Deleterious	Disease causing	4 of 4
c.5349G > A	p.Met1783Ile	Tolerated	Deleterious	Probably damaging	Neutral	Disease causing	3 of 4

The results from MDS and *in silico* prediction provide strong evidence for the enrichment of ethnic-specific deleterious variants in the unclassified variants.

### Pathogenic Variants and Prevalence

In the general population, we identified eight *BRCA* pathogenic and likely pathogenic variants, two in *BRCA1* with two carriers and six in *BRCA2* with six carriers. The eight pathogenic variant carriers in the 1,517 general individuals represent the prevalence of 0.53% *BRCA* pathogenic variants (0.13% in *BRCA1* and 0.40% in *BRCA2*) in Taiwanese population. The higher prevalence of *BRCA2* than *BRCA1* is consistent with the pattern in other Asian ethnic populations (Bhaskaran et al., 2020). With 0.53% prevalence, there are estimated 126,140 *BRCA* pathogenic variant carriers (30,940 in *BRCA1* and 95,200 in *BRCA2*) estimated in the Taiwanese population of 23.8 million or one *BRCA* pathogenic variant carrier in every 189 Taiwanese individuals. In the cancer cohort of 2,665 cases, we identified 74 *BRCA* pathogenic variants, 31 in *BRCA1* with 40 carriers (2.1% in 1,880 cases) and 43 in *BRCA2* with 61 carriers (2.5% in 2,417 cases), resulting in the prevalence of 3.8% in the Taiwanese cancer cohort of breast/ovarian cancer. Five pathogenic variants (two in *BRCA1* and three in *BRCA2*) were present only in general population with five carriers, and three *BRCA2* pathogenic variants were present in both general population and the cancer cohort with six carriers. *BRCA2* c.5164_5165delAG was a known pathogenic variant in Chinese cancer patients (Kwong et al., 2012). This pathogenic variant was present in the cancer cohort with 10 carriers but not in the general population. This variant is a potential founder mutation in the Taiwanese population and need to be validated by the haplotype test (Table 4).

**TABLE 4:** *BRCA* pathogenic and likely pathogenic variants identified in Taiwanese population.

Variant impact	cDNA	Protein	Mutation type	Cases	Carrier	References^a^
*BRCA1*						
Pathogenic	c.66dupA	p.Glu23Argfs*17	Frameshift insertion	120	1	15
Pathogenic	c.303 T > A	p.Tyr101Ter	Stopgain	28	1	14
Pathogenic	c.470_471delCT	p.Ser157Ter	Stopgain	133	1	10
Pathogenic	c.726delT	p.Ser242Argfs*4	Frameshift deletion	99	1	9
Pathogenic	c.928C > T	p.Gln310Ter	Stopgain	271	3	3,14,15
Pathogenic	c.981_982delAT	p.Cys328Ter	Frameshift deletion	1,517	1	TWB
Pathogenic	c.1361delG	p.Ser454Ilefs*20	Frameshift deletion	161	2	10.14
Pathogenic	c.1934delC	p.Ser645Leufs*5	Frameshift deletion	133	1	10
Pathogenic	c.2188G > T	p.Glu730Ter	Stopgain	99	1	9
Pathogenic	c.2393delC	p.Pro798Glnfs*4	Frameshift deletion	68	1	11
Pathogenic	c.2679_2682delGAAA	p.Lys893Asnfs*105	Frameshift deletion	133	1	10
Pathogenic	c.3083delG	p.Arg1028Leufs*19	Frameshift deletion	28	1	14
Pathogenic	c.3228_3229delAG	p.Gly1077Alafs*7	Frameshift deletion	253	2	10.15
Pathogenic	c.3257 T > G	p.Leu1086Ter	Stopgain	120	1	15
Pathogenic	c.3472delG	p.Glu1158Lysfs*1	Frameshift deletion	68	1	11
Pathogenic	c.3607C > T	p.Arg1203Ter	Stopgain	201	3	10.11
Pathogenic	c.3637G > T	p.Glu1213Ter	Stopgain	68	1	11
Pathogenic	c.3644_3648delACTTA	p.Asn1215Ilefs*1	Frameshift deletion	480	1	12
Pathogenic	c.3770_3771delAG	p.Glu1257Glyfs*8	Frameshift deletion	253	2	10.15
Pathogenic	c.3858_3861delTGAG	p.Ser1286Argfs*19	Frameshift deletion	99	1	9
Pathogenic	c.4356delA	p.Ala1453Glnfs*1	Frameshift deletion	120	1	15
Pathogenic	c.4678_4679delGG	p.Gly1560Asnfs*12	Frameshift deletion	480	2	12
Pathogenic	c.5030_5033delCTAA	p.Thr1677Ilefs*1	Frameshift deletion	36	1	8
Pathogenic	c.5075-1G > A	—	Splice site	480	1	12
Pathogenic	c.5211_5212delAG	p.Gly1738Argfs*90	Frameshift deletion	99	1	9
Pathogenic	c.5332+1G > A	—	Splice site	167	2	9.11
Pathogenic	c.5335delC	p.Gln1779Asnfs*13	Frameshift deletion	36	1	8
Pathogenic	c.5470_5477delATTGGGCA	p.Ile1824Aspfs*2	Frameshift deletion	133	1	10
Pathogenic	c.5536C > T	p.Gln1846Ter	Stopgain	36	1	8
Likely pathogenic	c.122 A > G	p.His41Arg	Nonsynonymous SNV	658	1	13
Likely pathogenic	c.5072C > A	p.Thr1691Lys	Nonsynonymous SNV	36	1	8
Likely pathogenic	c.5288G > A	p.Gly1763Glu	Nonsynonymous SNV	1,517	1	TWB
Likely pathogenic	c.5396C > A	p.Thr1799Asn	Nonsynonymous SNV	120	1	15
*BRCA2*						
Pathogenic	c.-7_9del16	—	Frameshift deletion	480	1	12
Pathogenic	c.469_470delAA	p.Lys157Valfs*24	Frameshift deletion	480	2	12
Pathogenic	c.631G > C	p.Val211Leu	Nonsynonymous SNV	133	1	10
Pathogenic	c.750_753delGACA	p.Asp252Valfs*23	Frameshift deletion	1,517	1	TWB
Pathogenic	c.755_758delACAG	p.Asp252Valfs*23	Frameshift deletion	480	1	12
Pathogenic	c.773_774delAA	p.Glu260Serfs*13	Frameshift deletion	1,616	2	9,TWB
Pathogenic	c.774_775delAA	p.Glu260Serfs*13	Frameshift deletion	658	1	13
Pathogenic	c.857C > G	p.Ser286Ter	Stopgain	480	1	12
Pathogenic	c.1036_1037delAA	p.Asn346Profs*9	Frameshift deletion	68	1	11
Pathogenic	c.1058C > T	p.Ser353Leu	Nonsynonymous SNV	1,517	1	TWB
Pathogenic	c.1765_1766delAA	p.Lys589Valfs*6	Frameshift deletion	1,517	1	TWB
Pathogenic	c.2095C > T	p.Gln699Ter	Stopgain	480	1	12
Pathogenic	c.2339C > G	p.Ser780Ter	Stopgain	99	1	9
Pathogenic	c.2442delC	p.Met815Trpfs*9	Frameshift deletion	516	2	2.12
Pathogenic	c.2754delC	p.Asn918Lysfs*41	Frameshift deletion	480	1	12
Pathogenic	c.2808_2811delACAA	p.Ala938Profs*20	Frameshift deletion	613	2	10.12
Pathogenic	c.2845delT	p.Tyr949Metfs*10	Frameshift deletion	36	1	2
Pathogenic	c.2990 T > G	p.Leu997Ter	Stopgain	480	1	12
Pathogenic	c.3109C > T	p.Gln1037Ter	Stopgain	600	4	12.15
Pathogenic	c.3322 A > T	p.Lys1108Ter	Stopgain	480	1	12
Pathogenic	c.4914dupA	p.Val1639Serfs*2	Frameshift insertion	480	1	12
Pathogenic	c.5141_5144delATTT	p.Tyr1714Cysfs*9	Frameshift deletion	480	1	12
Pathogenic	c.5164_5165delAG	p.Ser1722Tyrfs*3	Frameshift deletion	740	10	9,10,12,14
Pathogenic	c.5574_5577delAATT	p.Ile1859Lysfs*2	Frameshift deletion	133	1	10
Pathogenic	c.5621_5624delTTAA	p.Ile1874Argfs*33	Frameshift deletion	480	1	12
Pathogenic	c.6275_6276delTT	p.Leu2092Profs*6	Frameshift deletion	480	1	12
Pathogenic	c.6448delA	p.Val2151Phefs*16	Frameshift deletion	133	1	10
Pathogenic	c.6468_6469delTC	p.Gln2157Ilefs*17	Frameshift deletion	36	1	2
Pathogenic	c.6484_6485delAA	p.Lys2162Thrfs*12	Frameshift deletion	28	1	14
Pathogenic	c.6490C > T	p.Gln2164Ter	Stopgain	480	1	12
Pathogenic	c.6645delC	p.Ser2216Profs*12	Frameshift deletion	133	1	10
Pathogenic	c.6800C > A	p.Ser2267Ter	Stopgain	480	1	12
Pathogenic	c.7409dupT	p.Thr2471Hisfs*3	Frameshift insertion	133	1	10
Pathogenic	c.7567_7568delCT	p.Leu2523Glufs*14	Frameshift deletion	68	1	11
Pathogenic	c.7977-1G > T	—	Splice site	68	1	11
Pathogenic	c.8009C > T	p.Ser2670Leu	Nonsynonymous SNV	2,175	2	13,TWB
Pathogenic	c.8234dupT	p.Thr2746Aspfs*17	Frameshift insertion	480	1	12
Pathogenic	c.8243G > A	p.Gly2748Asp	Nonsynonymous SNV	133	1	10
Pathogenic	c.8323delA	p.Met2775Cysfs1*	Frameshift deletion	133	1	10
Pathogenic	c.8485C > T	p.Gln2829Ter	Stopgain	480	1	12
Pathogenic	c.8488-1G > A	—	Splice site	99	1	9
Pathogenic	c.8531_8532delAA	p.Glu2846Glyfs2*1	Frameshift deletion	133	1	10
Pathogenic	c.8961_8964delGAGT	p.Ser2988Phefs*11	Frameshift deletion	480	1	12
Pathogenic	c.9227delG	p.Gly3076Aspfs6*	Frameshift deletion	480	1	12
Pathogenic	c.9739C > T	p.Gln3247Ter	Stopgain	2,175	2	13,TWB
Likely pathogenic	c.3883C > T	p.Gln1295Ter	Stopgain	480	1	12

a Supplementary Table S1 lists each of the references. TWB: Taiwan Biobank.

## Discussion

It is well known that the number of benign variants is higher in the general population than in the cancer cohort, and the number of pathogenic mutations is higher in the cancer cohort than in the general population. Our current study aimed to obtain the detailed variant information including position, frequency, classification, and ethnic specificity in the Taiwanese healthy population and the cancer cohort in order to understand the genetic basis of *BRCA* variation in the population and to develop a precise reference to guide clinical applications.

Taiwanese population has its unique genetic features in reflecting its evolutionary and admixture history (Chen et al., 2016). With a population size of 23.8 million, *BRCA* variation information provides a unique source to understand its genetic variation in adaptation to the unique environment and the pathogenic variation causing cancer risk in the population. Data from our study provide an overview for *BRCA* variation and pathogenicity in this population, and further confirms the highly ethnic-specific nature of *BRCA* variation in eastern Asian population (Bhaskaran et al., 2020).

The availability of *BRCA* variant data from both general population and the cancer cohort allows comparison of the similarity and differences of *BRCA* variation between the two groups with the same ethnic background under the same geological environment. The higher rate of *BRCA* variation in its general population over other ethnic populations may reflect the rapidly evolving *BRCA* in Taiwanese population for better adaptation in Taiwan’s natural environment (Chen et al., 2016). This could be a factor contributing to higher prevalence of pathogenic variation in Taiwanese general population by increased probability of generating more pathogenic variants. The prevalence of 0.53% of pathogenic variation in the general Taiwanese population is the highest in Asian ethnic populations, comparing to 0.26% in Japanese (Momozawa et al., 2018), 0.29% in southern Chinese (Qin et al., 2020), 0.38% in mainland Chinese (Dong et al., 2020), and 0.39% in Malaysia (Wen et al., 2018), and has reached the same level of 0.53% as in Caucasian populations (Kurian et al., 2019). One *BRCA* pathogenic variant carrier in every 189 Taiwanese individuals represents a serious threat for public health in Taiwanese population, justifying the inclusion of *BRCA*-related cancer diagnosis, treatment, and prevention in the healthcare system in Taiwan. Considering its impact on population health, further confirmation of the result with a larger sample size will be necessary to validate the observations. The prevalence of 3.8% in the cancer cohort was lower than that in other ethnic cancer patient groups, such as 5.4% in Caucasians (Sun et al., 2017) and 5.3% in mainland Chinese (Bhaskaran et al., 2019).

All the pathogenic variants identified in Taiwanese population are present in public *BRCA* databases. Similar situation exists for the pathogenic variants identified in other Asian populations (Bhaskaran et al., 2019; Bhaskaran et al., 2020; Dong et al., 2020; Qin et al., 2020; Zhang et al., 2020). In the meantime, 44.1% of the *BRCA* variants identified in Taiwanese population remain as novel, VUS, and unclassified variants. Our *BRCA* study across multiple ethnic Asian populations also showed that 30–50% of variants present in each population were novel, VUS, and unclassified variants. The distribution patterns of pathogenic and unclassified variants seem to suggest that pathogenic variants are universally shared between human populations, whereas non-pathogenic variants are largely ethnic specific. However, such assumption does not have a biological sense. Considering that *BRCA* variation is highly ethnic specific and a large portion of the *BRCA* variants identified in ethnic population remain unclassified, it will be logical to consider that ethnic-specific pathogenic variants should also exist, and these are likely enriched within the unclassified variants. The pathogenic variants highly shared between the human populations represent the common pathogenic variants inherited from their common ancestors. They are identifiable by referring to the current well-annotated *BRCA* pathogenic data predominately derived from Caucasian populations (Rebbeck et al., 2018; Bhaskaran et al., 2019). As these reference databases lack the pathogenic variant data from the non-Caucasian populations, the ethnic-specific pathogenic variants in the non-Caucasian populations are not identifiable by referring to these databases. The ethnic-specific pathogenic variants can be highly enriched within the ethnic-specific novel, VUS, and unclassified variants, as evidenced from our MDS and *in silico* analyses. However, it remains a challenge in cancer genetic study to develop extensive ethnic-specific pathogenic variant references.

In summary, the data generated from the study provide a comprehensive view for *BRCA* variation in the Taiwanese population and a reference for clinical applications in *BRCA*-related cancer in the Taiwanese population.

## Materials and Methods

### Data Sources

Studies were selected by the following inclusion criteria: 1) cancer patients should be pathologically confirmed, 2) germline variants in *BRCA1/2* should be genotyped, and 3) studies on nonhuman or cell line were excluded. Thoroughly searching PubMed and Google Scholar using the keywords such as “*BRCA1*” “*BRCA2*” “Taiwan” “Taiwanese” and “cancer predisposition” we identified 15 publications reporting the *BRCA* data from Taiwanese cancer patients between 1997 and 2020 (Liu et al., 1997; Li et al., 1999; Wang et al., 2000; Chen et al., 2003; Lin et al., 2003; Chen et al., 2005; Chang et al., 2006; Kuo et al., 2012; Chao et al., 2016; Lin et al., 2016; Sung et al., 2017; Wang et al., 2018; Lin et al., 2019; Chao et al., 2020; Lin et al., 2020). Searching Taiwan Biobank (https://taiwanview.twbiobank.org.tw/index; accessed December 15, 2020) using the keywords “*BRCA1*” and “*BRCA2*” we obtained the *BRCA* variation data derived from Taiwanese general population.

### Data Analysis

The following details were extracted from the filtered publications, including first author, year of publication, *BRCA* variants, mutation type of variants, study population, and the number of cases in the study. We standardized the collected *BRCA* variation data following the Human Genome Variation Society (HGVS) guidelines (den Dunnen et al., 2016). The following reference sequences were used for the mapping analysis: *BRCA1*: cDNA NM_007294.3, protein NP_009225.1, and genome hg19 NC_000017.10; *BRCA2*; cDNA NM_000059.3, protein NP_000050.2, and genome hg19 NC_000013.10. We annotated the variants using the ANNOVAR program (Wang et al., 2010). The population frequency was referred to East Asian variants (EAC) from the 1,000 Genome Project (Fairley et al., 2020), the Exome Aggregation Consortium (ExAC) (Lek et al., 2016), and the Genome Aggregation Database (gnomAD) (Karczewski et al., 2020). The variants were compared with the following two *BRCA* databases: the BRCA Exchange Database (BED, http://brcaexchange.org, accessed December 15, 2020) and ClinVar (http://www.ncbi.nlm.nih.gov/clinvar/, accessed December 15, 2020). The variants present in *BRCA* databases were classified as known variants by referring to the existing classification of pathogenic, likely pathogenic, uncertain significance, likely benign, and benign. The classes for those variants not present in existing *BRCA* databases were predicted using the InterVar program with default parameters (Li and Wang, 2017). The Fujianese *BRCA* variants were extracted from the whole genome sequences of Fujian individuals (Yang et al., 2020).

### Molecular Dynamics Simulations and *in silico* Prediction

We utilized molecular dynamics simulations (MDS) to measure the impact of the four BRCA1 unclassified variants in the Taiwanese population (c.5068A > C p.Lys1690Gln, c.5347A > C p.Met1783Leu; c.5347A > G p.Met1783Val; and c.5349G > A p.Met1783Ile) on the stability of the *BRCA1* BRCT structure. The MDS system was developed for *BRCA1* BRCT variant classification as described in details (Sinha and Wang, 2020). In brief, the process included two major steps: 1) modeling mutant structure. Using the wild-type BRCT structure as the template, each mutant structure was constructed using the Modeller program (*version 9.22,* UCSF, CA, United States), and further evaluated using the PROCHECK (Sippl, 1993) and PROSA (Wiederstein et al., 2007) programs following the instructions; 2) analyzing the impact of variants on BRCT structural stability by using MDS (Karplus, 2002). Using the wild-type structure as the reference, MDS analyzes the trajectory of the mutant structure over a time period through multiple parameters including RMSD (root mean square deviation) to measure the average deviation in the backbone of Cα trace (Dong et al., 2018), RMSF (root mean square fluctuations) to measure the residue flexibility of the structure (Benson et al., 2012), Rg (radius of gyration) to measure the distance of the atoms of the structure from its center of gravity and axis for the compactness of each structure (Daidone et al., 2003), SASA (solvent accessible surface area) to measure the surface accessibility (Sheu et al., 2003), NH-bond (number of hydrogen bonds) to measure the overall change in the compactness of the mutant structures, and covariance analysis to compare the overall protein motions (Amadei et al., 1993).

Four *in silico* prediction methods of SIFT (Kumar et al., 2009), Polyphen2_HDIV (Adzhubei et al., 2010), LRT (Chun et al., 2009), and MutationTaster (Schwarz et al., 2010) were used to predict the deleteriousness for the four unclassified variants in BRCT repeats following the default setting in each method.

### Statistical Analysis

A chi-square test was used to compare the differences of *BRCA* variant data between different populations using SPSS (version 26.0, IBM, NY, United States). A *p* value lower than 0.05 was considered as statistically significant.

## Data Availability

The datasets presented in this study can be found in online repositories. The names of the repository/repositories and accession number(s) can be found in the article/Supplementary Material.
